# Cellular immune response to human influenza viruses differs between H1N1 and H3N2 subtypes in the ferret lung

**DOI:** 10.1371/journal.pone.0202675

**Published:** 2018-09-07

**Authors:** Kathryn A. Ryan, Gillian S. Slack, Anthony C. Marriott, Jennifer A. Kane, Catherine J. Whittaker, Nigel J. Silman, Miles W. Carroll, Karen E. Gooch

**Affiliations:** National Infection Service, Public Health England, Porton Down, Wiltshire, United Kingdom; The University of Chicago, UNITED STATES

## Abstract

Seasonal influenza virus infections cause yearly epidemics which are the source of a significant public health burden worldwide. The ferret model for human influenza A virus (IAV) is widely used and has several advantages over other animal models such as comparable symptomology, similar receptor distribution in the respiratory tract to humans and the ability to be infected with human isolates without the need for adaptation. However, a major disadvantage of the model has been a paucity of reagents for the evaluation of the cellular immune response. Investigation of T-cell mediated immunity in ferrets is crucial to vaccine development and efficacy studies. In this study we have used commercially produced antibodies to ferret interferon gamma (IFN-γ) allowing us to reliably measure influenza-specific IFN-γ as a marker of the cellular immune response using both enzyme-linked immunospot (ELISpot) and enzyme-linked immunosorbent (ELISA) techniques. Here we demonstrate the application of these tools to evaluate cellular immunity in ferrets infected with clinically relevant seasonal H1N1 and H3N2 IAV subtypes at equivalent doses. Using small heparinised blood samples we were able to observe the longitudinal influenza-specific IFN-γ responses of ferrets infected with both seasonal subtypes of IAV and found a notable increase in influenza-specific IFN-γ responses in circulating peripheral blood within 8 days post-infection. Both seasonal strains caused a well-defined pattern of influenza-specific IFN-γ responses in infected ferrets when compared to naïve animals. Additionally, we found that while the influenza specific IFN-γ responses found in peripheral circulating blood were comparable between subtypes, the influenza specific IFN-γ responses found in lung lymphocytes significantly differed. Our results suggest that there is a distinct difference between the ability of the two seasonal influenza strains to establish an infection in the lung of ferrets associated with distinct signatures of acquired immunity.

## Introduction

Influenza is a significant global health problem with 5–15% of the population infected each year, leading to approximately 200,000 to 500,000 influenza attributed deaths [1, 2]. Influenza has an RNA genome and this allows the virus to mutate rapidly leading to antigenic drift and shift. Antigenic drift occurs via the gradual change of viral surface epitopes, leading to yearly epidemics, whilst antigenic shift is caused by the reassortment of genomes between two or more strains of influenza, which can ultimately lead to new and potentially pandemic strains. Influenza A virus (IAV) infects humans and is split into subtypes based on the neuraminidase (NA) and haemagglutinin (HA) surface glycoproteins found on the virus. There are currently two IAV subtypes circulating in the human population; H1N1 and H3N2. The H1N1 virus originated from the 2009 influenza pandemic caused by a reassortment of human, swine and bird type A influenza[3], while the current seasonal H3N2 strain originated from the 1968 Hong Kong pandemic when genomic exchange of RNA occurred between human and avian viruses[4]. Vaccination is the most efficient method to protect the population from influenza; therefore understanding seasonal influenza strains is important since they dictate annual vaccine strategies.

Two types of vaccine are currently used to combat seasonal influenza; the trivalent inactivated vaccines (TIV) which elicit a mainly humoral response and the live attenuated influenza vaccine (LAIV) which elicits a protective mucosal immune response without causing clinical disease[5]. Both need to be reformulated annually due to their strain-specific immune responses. Much current research is directed at developing novel, more universally protective vaccines, and animal models are required to facilitate assessment of novel vaccine immunogenicity and efficacy. An ideal universal vaccine would confer long term protection to influenza viruses with broad immune coverage including antibodies and T-cells In practice this has not been the case for the current inactivated vaccine, especially during seasons where a mismatch between circulating and current strains occur[6], strengthening the need for a universal vaccine.

There are a range of animal models that have been used to study influenza. Ferrets have been used extensively to study influenza pathogenesis [7–10], transmission [7, 11–14], vaccine and antiviral efficacy [15–17] and remain the gold standard animal model for IAV. Ferrets are highly susceptible to both human and avian influenza strains [2], they have comparable clinical signs and disease outcomes to humans when infected with influenza viruses as well as having similar sialic acid receptors in their respiratory tracts when compared to humans. Not unlike humans, ferrets infected with seasonal H1N1 and H3N2 strains produce mild clinical symptoms with widespread infection of the upper respiratory tract due to the viruses high affinity for alpha 2,6- linked sialic acid receptors while tissues in the lower respiratory tract are decreasingly affected [8]. The evaluation of the cell mediated immune response in ferrets is exceptionally important for modelling human disease. It has been shown that T-cells recognise epitopes derived from conserved internal proteins of IAV and crucially, human T-cells do not distinguish between IAV sub-types due to conservation of specific structural proteins[18]. This is significant as it may provide a key to a universal vaccine that could be used to vaccinate against multiple IAV strains over several seasons negating the requirement to formulate a new vaccine each season. Moreover, T-cell mediated immunity has recently been shown to be a significant correlate of protection in humans [18–20] therefore it is important to develop key techniques for the ferret model to provide a well characterised animal model available for study of T-cell mediated protective immunity in order to help us identify new correlates of protection.

Recent ferret studies have established a number of techniques and reagents that enable the identification and quantifications of key cell types and cytokines involved in antigen specific T-cell responses following influenza infection [21–23]. One of these cytokines is the multipotent Interferon gamma (IFN-γ) which plays a key role in the development of innate and adaptive immune response [24–27] and is widely accepted as a marker of the adaptive immune response IFN-γ is produced by CD4+ Th1 cells, most CD8+ cells, and NK cells, is known to be an important contributor to antiviral immunity to influenza [28] and is able to control viral infection and induce inflammatory damage [29]. Previous ferret studies have identified that IFN-γ T-cell responses allow a more precise assessment of the vaccine induced protection level [27]. Studies have also looked at cell populations of CD4+ and CD8+ T-cells, using influenza-specific IFN-γ responses to characterise leukocyte composition and antigen-specific T-cell responses in key lymphoid tissue following influenza infection in ferrets using an enzyme-linked immunospot (ELISpot) method [21] as well as daily tracking of peripheral blood leukocytes in infected ferrets using a flow cytometric technique [16]. These studies infect ferrets with relatively high doses of influenza virus and fail to provide a comparison of the cellular immune response between strains. In addition they are unable to provide an illustration of the ongoing influenza-specific IFN-γ responses as a marker of the cellular immune response in the ferret during infection.

Despite the numerous advantages of the ferret model, ferrets are a relatively small animal and therefore it is not possible to collect the large volumes of blood required for some cellular immune evaluation methods without termination. This, coupled with the previous lack of available reagents has significantly hampered efforts to study the cellular immune response to influenza in ferrets. To address this, we performed a longitudinal time course assessing influenza- specific IFN-γ responses to two clinically relevant subtypes of seasonal influenza, H1N1 (A/California/04/2009) and H3N2 (A/Perth/16/2009) negating the need to sacrifice the animals. This allowed us to comprehensively look at the influenza-specific IFN-γ response as a marker of the cellular immune response in the periphery, and go on to compare and contrast this to the influenza-specific IFN-γ responses seen in the lung and peripheral blood mononuclear cells (PBMCs) upon study conclusion.

## Methods

### Virus

Influenza A/California/04/2009 (H1N1) and A/Perth/16/2009 (H3N2) were propagated in Madin-Darby Canine Kidney (MDCK) cells obtained from The European Collection of Authenticated Cell Cultures (ECACC, Porton Down, United Kingdom). The identity of both viruses was confirmed by sequencing the HA and NA genes. Virus titres were determined by plaque assay on MDCK cells under an agar overlay, followed by staining with crystal violet.

### Ferrets

Adult (4–6 months) ferrets (*Mustela putorious fura)* from Highgate Farm, UK, confirmed seronegative for influenza A/California/04/2009 (H1N1) and A/Perth/16/2009 (H3N2) by haemagglutination inhibition (HAI) assay, were used for these studies. Ferrets were weighed once daily at approximately the same time. Appetite, sneezing, nasal discharge, diarrhoea and activity level were monitored twice daily and were recorded as present or absent. Activity levels were scored from 0–2 (0 = normal, 1 = reduced activity and 2 = inactive) A score was generated by summing the number of observations of each sign over the 14 days post-challenge. Nasal washes were obtained using 2ml PBS. Ferrets were allocated into five study groups as illustrated in **Table 1**. The animal study described was scrutinized and approved by the Animal Welfare and Ethical review Body of Public Health England (Porton), as required by the UK Home Office Animals (Scientific Procedures) Act, 1986.

**Table 1:** Experimental animal groups.

Group	Challenge Virus	Number of Animals	Nasal Wash Sample Days	Whole Blood Sample Days
1	A/California/04/2009	4	1–2, 5, 8, 11, 14	N/A
2	A/California/04/2009	6	1–8, 11, 14	-7, -1, 2, 5, 8, 11, 14
3	A/Perth/16/2009	3	1–8, 11, 14	-7, -1, 2, 5, 8, 11, 14
4	A/Perth/16/2009	6	1–8, 11, 14	-7, -1, 2, 5, 8, 11, 14
5	PBS	6	1–8, 11, 14	-7, -1, 2, 5, 8, 11, 14

A total of 25 ferrets were distributed across 5 groups. Animals were infected with a low dose (10^2^ pfu/ferret) of each virus. A naïve group of 6 animals was given PBS. All inoculations were performed intranasally with 0.2ml of fluid.

### Virus infection of ferrets

Influenza stock was diluted in PBS to provide a challenge dose of 10^2^ pfu/ferret, confirmed using back titration by plaque assay. Animals were sedated by intramuscular injection using ketamine/xylazine given at a dose of 0.25ml/kg bodyweight prior to intranasal administration of 0.2ml of challenge virus, 0.1ml per nostril. Naïve animals were sedated as described, prior to intranasal administration of 0.2ml of PBS, 0.1ml per nostril.

### Isolation of peripheral blood mononuclear cells

Fresh heparin anti-coagulated blood was layered on room temperature Histopaque 1083 (Sigma- Aldrich, Dorset, United Kingdom) in 15ml ACUSPIN^TM^ tubes (Sigma-Aldrich, Dorset, United Kingdom) and a density separation carried out at 800g for 20 minutes. The buffy coats containing lymphocytes were collected and washed with medium (R2) consisting of RPMI 1640 medium (Sigma-Aldrich, Dorset, United kingdom) with the addition of L-glutamine (2mM)(Sigma-Aldrich, Dorset, united Kingdom), 0.05mM 2-mercaptoethanol (Invitrogen, Paisley, United Kingdom), 25mM HEPES buffer (Sigma-Aldrich, Dorset, United Kingdom), and 2% heat inactivated foetal bovine serum (Sigma-Aldrich, Dorset, United Kingdom), cells were pelleted by centrifugation at 400g for 10 minutes.

### Isolation of lung mononuclear cells

Whole lungs were removed from each ferret. The lungs were dissected into small pieces and placed into a 25ml solution of collagenase (715 collagenase units/ml) (Sigma-Aldrich, Dorset, United Kingdom) and DNase (350 DNase units/ml)(Sigma-Aldrich, Dorset, United Kingdom). Lungs were vigorously shaken whilst incubating at 37^°^C for 1 hour. Partially digested lung tissue was then placed into gentleMACS C-tubes and dissociated using a gentleMACS Tissue Dissociator (Miltenyi Biotec, Surrey, United Kingdom). The tissue solution was passed through two cell sieves (100μm then 70μm) and then layered on room temperature Histopaque 1083 (Sigma- Aldrich, Dorset, United Kingdom). A density separation was carried out at 400g for 30 minutes. The buffy coats containing lymphocytes were collected and washed with R2 medium by pelleting cells by centrifugation at 400g for 10 minutes.

### Red blood cell removal using ACK lysis buffer

Red blood cells were lysed from PBMC and lung MNC preparations by re-suspending cell pellets in 5ml of ACK Lysing Buffer (Gibco, ThermoFisher Scientific, United Kingdom). The cells were incubated at room temperature with gentle agitation for 5 minutes. The ACK Lysing Buffer was inactivated by the addition of an excess of R2 medium. Cells were pelleted at 400g for 5 minutes to remove lysis buffer. If lysis of the red blood cells was incomplete the treatment was repeated.

### Viable cell counts

Viable cells in nasal washes, PBMCs and lung MNCs were counted using a NucleoCounter®NC-200^TM^ (ChemoMetec, Allerod, Denmark).

### Cryopreservation of cells

Cells were pelleted by centrifuging at 400g for 5 minutes. Cells were then re-suspended in an appropriate volume of cryomedia (90% FCS + 10% DMSO) allowing the cells to be frozen in liquid nitrogen in 1ml aliquots at a concentration of 3 x10^6^ to 1.3 x 10^7^ cells/ml.

### Interferon-gamma (IFN-γ) ELISpot assay

An IFN-γ ELISpot assay was performed with PBMC and lung MNC to determine the production capacity of influenza-specific T cells in PBMC and Lungs using a Ferret IFN-γ kit (Mab Tech, Nacka. Sweden). PBMC and lung MNC were defrosted into pre-warmed medium (R10) consisting of RPMI 1640 medium (Sigma-Aldrich, Dorset, United kingdom) with the addition of 2mM L-glutamine (Sigma-Aldrich, Dorset, United Kingdom), 0.05mM 2-mercaptoethanol (Invitrogen, Paisley, United Kingdom), 25mM HEPES buffer (Sigma-Aldrich, Dorset, United Kingdom), 10% heat inactivated foetal bovine serum (Sigma-Aldrich, Dorset, United Kingdom), and benzonase (Novogen, Merck, Darmstadt, Germany). Cells were rested for 2 hours prior to use. PBMC and lung MNC were assessed for responses to A/California/07/2009 (H1N1) and A/Perth/16/2009 (H3N2). Both viruses were used at a MOI of 0.08 to re-stimulate the PBMCs and lung MNCs. Viruses were egg grown, therefore, egg allantoic fluid was used as a negative control. Phorbol-12-myristate (100ng/ml; Sigma-Aldrich, Dorset, United Kingdom) and ionomycin (1μg/ml; Merck, Watford, United Kingdom) were combined and used as a positive control. Pre-coated (mAb MTF14) plates (Mab Tech, Nacka. Sweden) were used. 50,000 lung MNCs and 200,000 PBMCs were plated per well in 50μl of R10, with or without antigen, in duplicate and incubated overnight. Following culture, plates were washed and incubated for 2 hours with biotinylated anti IFN-γ IgG. Spots were developed by the addition of streptavidin-alkaline phosphatase and 5-bromo-4-chloro-3-indoly phosphate (BCIP)-Nitro Blue tetrazolium (NBT) substrate. Results from duplicate tests were averaged. Data were analysed by subtracting the mean number of spots in the cells and allantoic fluid control wells from the mean counts of spots in wells with cells and antigen.

### Quantification of influenza specific IFN-γ production by enzyme linked immunosorbent assay (ELISA)

Heparinised whole blood was diluted 1:10 with serum free RPMI 1640 medium and incubated with either A/California/07/2009 (H1N1) or A/Perth/16/2009 (H3N2), corresponding with the virus of infection. Phytohemagglutinin PHA-M (PHA) (Sigma-Aldrich, Dorset, United Kingdom) was used as a positive control and egg allantoic fluid was used as a negative control as viruses were egg grown. Blood was stimulated for 4 days at 37°C, after which plasma supernatants were collected and cryopreserved at -80°C. The Ferret IFN-γ ELISA Development Kit (ALP) (Mab Tech, Nacka. Sweden) was used to determine the quantity of IFNɣ secreted by cells in the blood in responses to influenza-specific stimulations. ELISA plates were read using the VersaMax ELISA Microplate Reader (Molecular Devices, Sunnyvale, CA, USA) with SoftMax^TM^ PRO software (Molecular Devices, Sunnyvale, CA, USA).

### Statistical analysis

Statistical analysis was performed with GraphPad Prism 7 (GraphPad Prism, GraphPad Software, La Jolla, CA). The Mann-Whitney Test was used to analyse significant difference between groups. P < 0.05 was considered significant.

## Results

### Clinical signs and virus shedding following low dose challenge with seasonal influenza strains

Ferrets were intranasally infected with a well characterised low dose of influenza (10^2^ plaque forming units (PFU) per ferret) of either A/California/04/2009 (H1N1)[17] or A/Perth/16/2009 (H3N2) (**Table 1**). This low dose intranasal challenge has previously been shown to infect 100% of inoculated ferrets infected with H1N1[17] and was therefore selected as the method of challenge.

**Fig 1:**
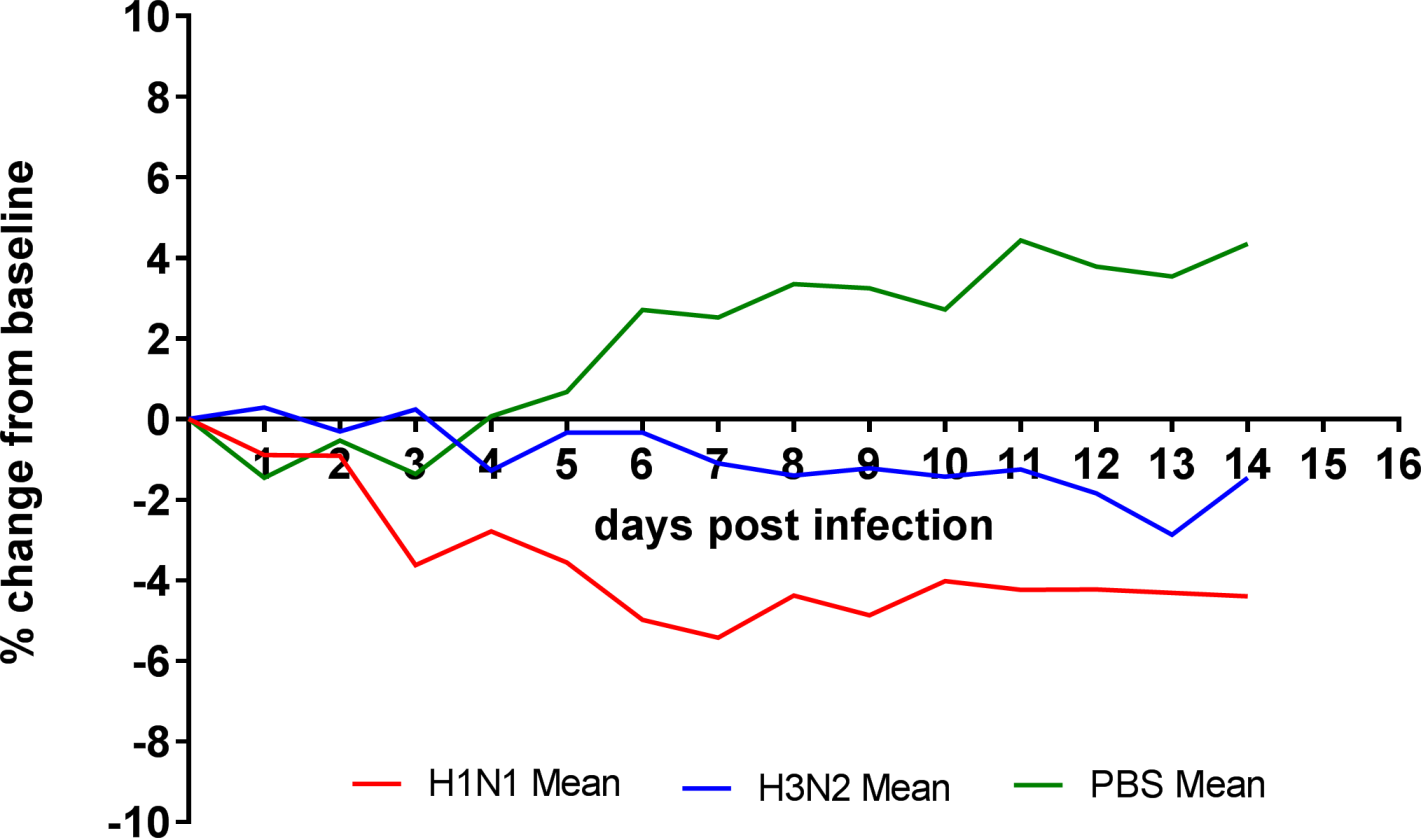
Clinical scoring. (a) Mean percentage weight change of ferrets infected with H1N1 (groups 1 and 2 combined, n = 10), H3N2 (groups 3 and 4 combined n = 9) and PBS (group 5, n = 6).

Disease progression was monitored up to 14 days post infection (dpi). Ferrets used as a naïve control (group 5) were mock infected intranasally with PBS and also monitored for 14 days. Weight loss from baseline was seen in all ferrets infected with influenza, in comparison mock infected ferrets gained weight (**Fig 1**).

**Fig 2:**
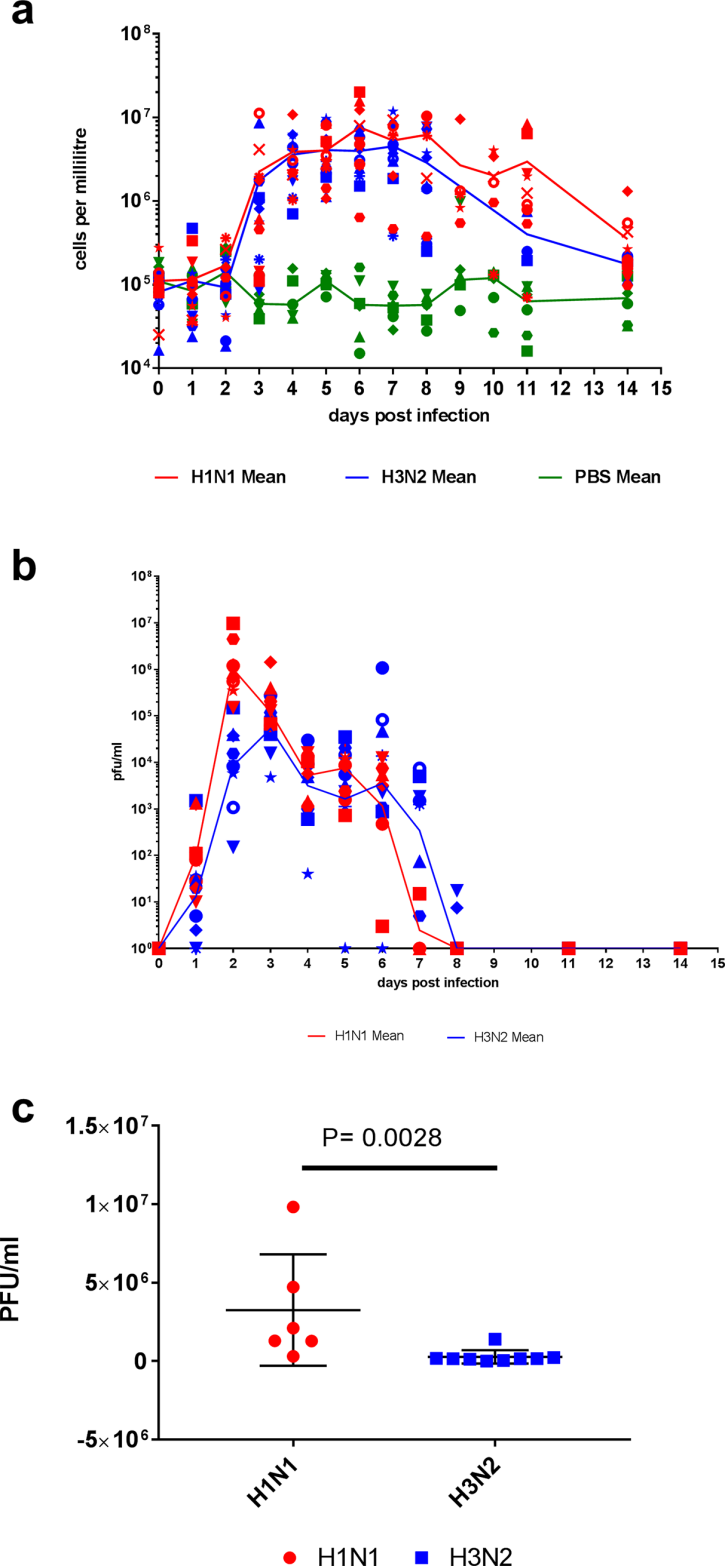
Nasal wash collection. Nasal washes were collected at +1 to +8 dpi, 11 dpi and 14 dpi for Groups 2–5. Group 1 had nasal washes taken at +1, +2, +5, +8, +11 and +14 dpi. (a) All nasal washes were counted to ascertain the number of cells being shed from each individual ferret at each time point (n = 25). (b) Nasal washes were subsequently plaque assayed to ascertain the titre of virus being shed from each individual ferret (n = 19) at these time points. (c) The area under the curve was calculated for groups 2 (n = 6), and 3 and 4 combined (n = 9). Group 1 was omitted from this statistical analysis as the nasal wash samples were not collected at the same time points as groups 2, 3 and 4. A Mann-Whitney test was performed and a statistically significant difference (P = 0.0028) was found between ferrets infected with H1N1 and H3N2.

All ferrets were monitored for sneezing, nasal discharge, inactivity, diarrhoea and loss of appetite. Animals infected with H1N1 showed more severe clinical signs compared to ferrets infected with H3N2. In particular, the H1N1-infected ferrets of groups 1 and 2 presented with sneezing, nasal discharge and inactivity (mean of 6.4, 1.7 and 6.8 observations per ferret, respectively). By contrast, the H3N2-infected ferrets of groups 3 and 4 presented with sneezing and nasal discharge only (mean 3.8 and 0.3 observations per ferret, respectively) (**Table 2**). An increase in nasal wash cell counts has previously been shown to correlate with successfully infected animals[17, 30] and therefore they were recorded daily from 1 dpi to 8 dpi, 11 dpi and 14 dpi in groups 2–5. Counts were recorded at 1, 2, 5, 8, 11 and 14 dpi for group 1 (**Table 1**). Nasal wash cell counts from all influenza infected ferrets began to increase between 2 and 4 dpi whilst mock infected ferrets did not increase over the 14 days (**Fig 2A**). No significant difference was found between the total amounts of cells shed between the groups infected with H1N1 and H3N2.

**Table 2:** Clinical sign observations.

Group	N	Total Sneezing	Total Nasal Discharge	Total Inactivity Score	Mean Score per Ferret
1+2	10	64	17	68	14.9
3+4	9	34	3	0	4.1
5	6	0	0	0	0

Total instances of clinical sign observations. N, number of ferrets per group.

In addition to cell counts, nasal wash samples were tested for the presence of replicating virus by plaque assay. Virus shedding in nasal washes began by 1 dpi for all 10 ferrets infected with H1N1 and 7 out of 9 ferrets infected with H3N2 (**Fig 2B**). Viral shedding in ferrets infected with H1N1 (groups 1 and 2) peaked at 2dpi, while viral shedding from ferrets infected with H3N2 (groups 3 and 4) peaked a day later at 3 dpi, followed by a decrease in viral load before a second smaller peak at 5 dpi (H1N1) and 6 dpi (H3N2). This smaller second peak was not found to be statistically significant; however it has been noted by others previously[31]. It is also worth noting that the second peak is not seen the nasal wash cell counts. Viral clearance (absence of live virus found in nasal wash) had occurred in all infected groups by 11 dpi. No virus was detected in the mock infected animals at any time. The peak shedding titre was found to have a strong positive correlation and significance (R +0.9781, P <0.0001) with the total virus shed from groups 1, 2, 3 and 4. Consequently, the more virus shed by an animal the higher the peak titre of virus found in nasal wash. Using area under the curve, which represents the total virus shed by each ferret, it was found that there was significantly (P 0.0028) more virus shed by ferrets infected with H1N1 (group 2) compared to ferrets infected with H3N2 (groups 3 and 4) (Fig 2C).

### Longitudinal time course of influenza-specific IFN-γ responses in the periphery/ IFN-γ response to challenge

**Fig 3:**
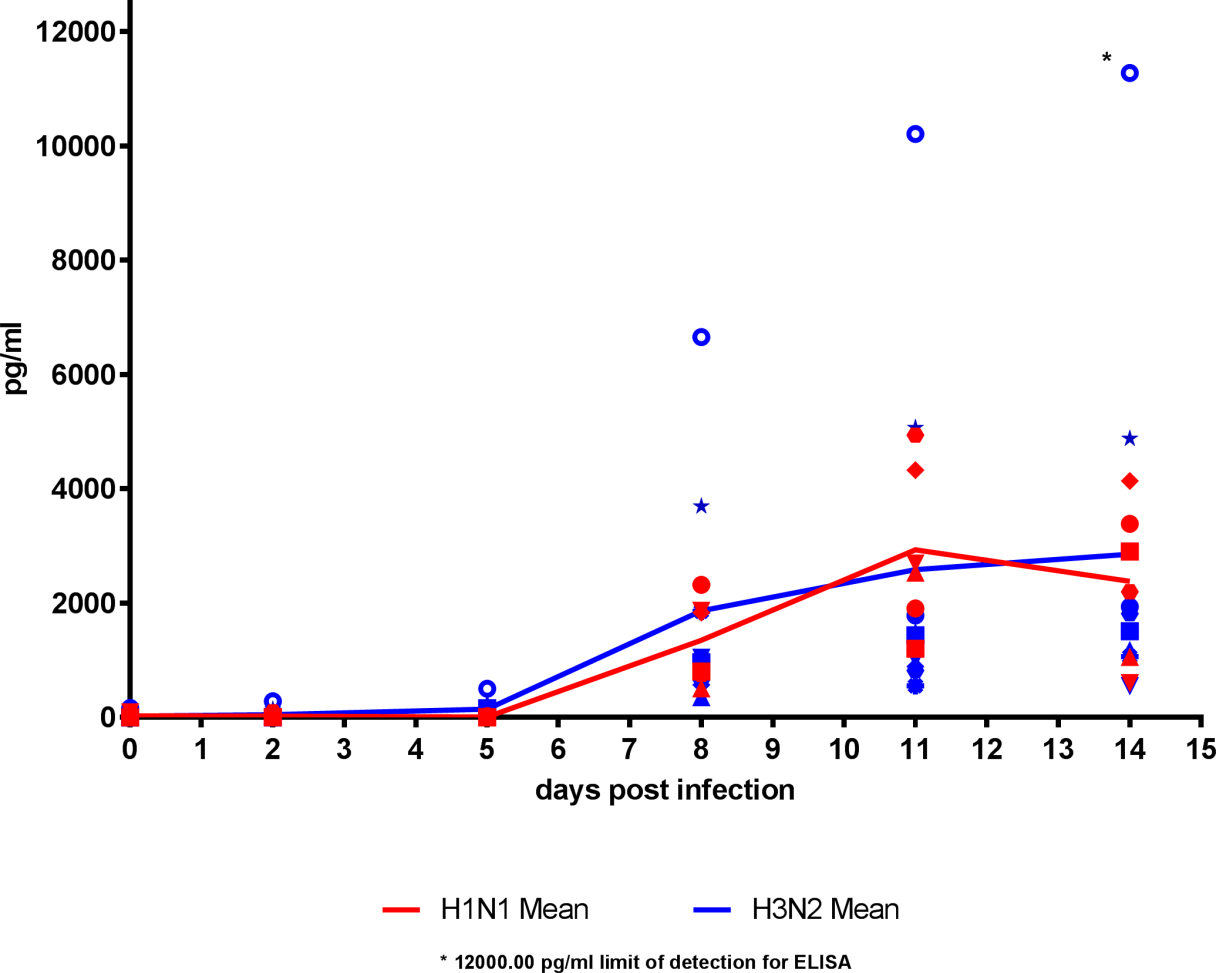
Quantification of influenza specific IFN-γ production by ELISA. Diluted whole blood samples were collected from groups 2 to 5 (n = 21) at -7, -1, +2, +5, +8, +11 and +14 dpi, stimulated with appropriate antigens and the supernatants harvested. Supernatants were used in the Ferret IFN-γ ELISA Development Kit (HRP). Influenza specific IFN-γ responses became detectable at 5 dpi with a peak at 11–14 dpi for all virus infected animals from groups 2 to 4 (n = 15). No responses were detected in mock ferrets (not shown, n = 6). 12000 pg/ml was the upper limit of detection for the ELISA.

Small volumes of heparinised whole blood were collected from groups 2, 3, 4 and 5 at days shown in **Table 1**to assess the influenza-specific IFN-γ responses in peripheral blood. This was done by stimulating diluted whole blood samples with homologous virus or appropriate controls as detailed in the methods section; secreted IFN-γ was detected by enzyme linked immunosorbant assay (ELISA). Samples from each ferret were assessed by ELISA and a longitudinal time course of influenza-specific IFN-γ responses was produced (**Fig 3**). Two samples were collected prior to infection (-7 and -1 dpi) to demonstrate that there were low influenza specific IFN-γ responses in the ferrets prior to infection. All ferrets were found to have responses below 270 pg/ml of IFN-γ in pre-infection samples. These responses were averaged for each animal and represented by the 0 dpi time point (**Fig 3**). Following infection with either H1N1 or H3N2, low level influenza-specific IFN-γ responses were detectable in ferrets at 5 dpi and increased at 8 and 11 dpi with responses peaking at 11 to 14 dpi. At 14 dpi, responses in the majority of ferrets appear to decrease while some others continue to increase. This variation could be due to the outbred nature of the ferrets. There was no significant difference found between the total influenza-specific IFN-γ produced in whole blood from ferrets infected with the H1N1 and H3N2 sub-types, nor was there a difference between the subtypes at any time-point.

### Cellular immune responses measured by ferret specific IFN-γ enzyme-linked immunospot assay (ELISpot)

**Fig 4:**
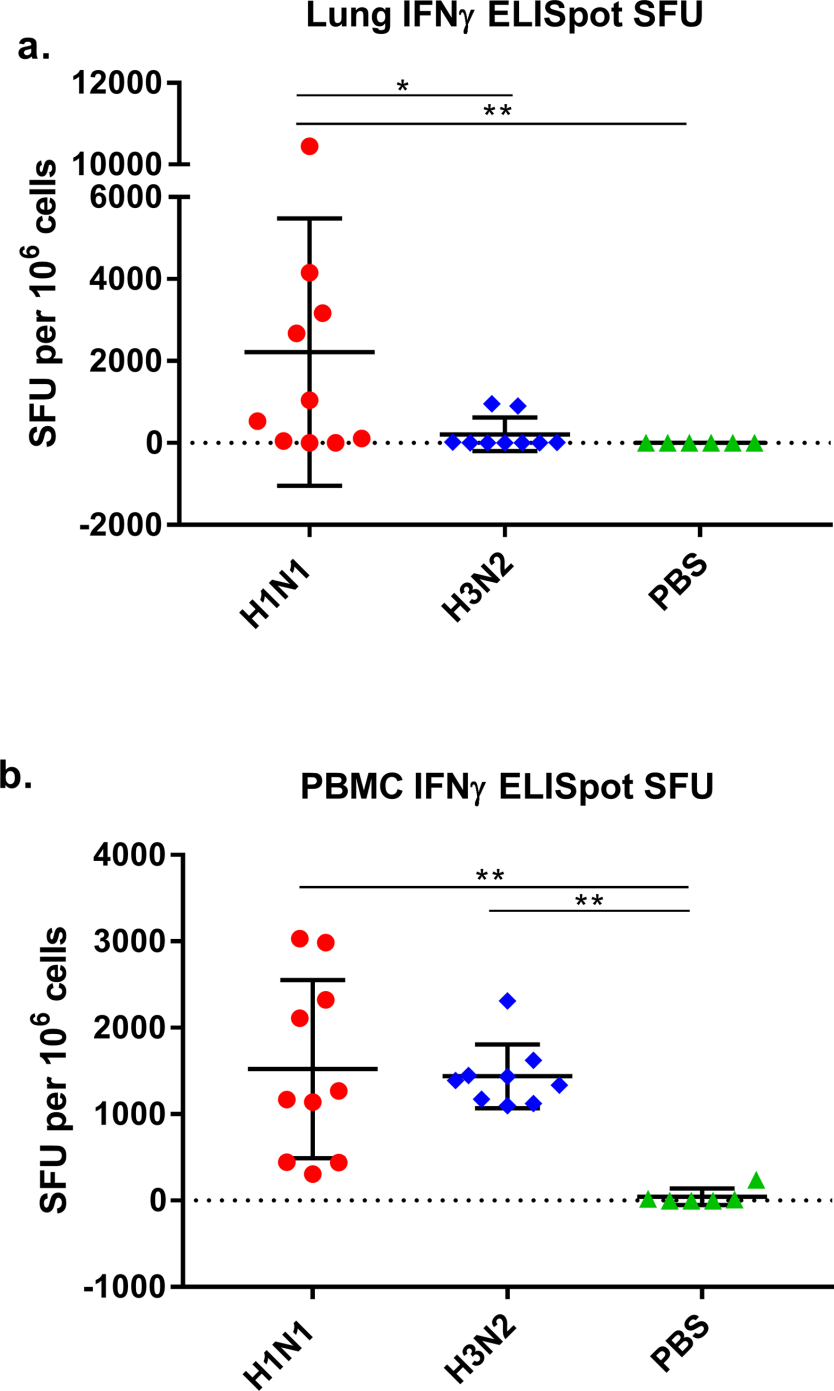
Cellular immune responses of ferrets infected with seasonal influenza. Lung MNCs (a) and PBMCs (b) were collected from all animals (n = 25) at 14 dpi. Results were normalised by subtracting the individual sample allantoic fluid control values from virus stimulated sample values. Influenza specific IFN-γ responses were quantified in lung MNC and PBMC samples from all ferrets. (a) Influenza specific IFN-γ responses in lung MNCs to corresponding viruses were seen in eight out of ten H1N1 infected ferrets. Only two of nine H3N2 infected ferrets were found to have influenza specific IFN-γ responses in lung MNCs, no responses were detected in remaining ferrets. (b) Influenza specific IFN-γ responses in PBMCs to corresponding viruses were seen in all virus infected ferrets. A very low influenza specific IFN-γ response was detected in the PBMCs of one ferret (less than those seen in the low H1N1 infected ferrets), none of the remaining PBMCs or lung MNCs of mock ferrets had any detectable influenza specific IFN-γ responses. The values measured for each ferret are plotted as spot forming units (SFU) per million cells. Bars show mean and standard deviation for each group.

Animals were culled at 14 dpi. Lymphocytes were isolated from whole lung (lung MNCs) and whole blood (PBMCs) and the frequency of IFN-γ secreting cells was quantified by ferret-specific INF-γ ELISpot. We found a significant difference in the number of cells producing influenza-specific IFN-γ in H1N1 infected animals compared to H3N2 infected animals (P 0.0306) (**Fig 4A**). The mean values of the H1N1 group was 2305 SFU while the mean value for the H3N2 group was 210 SFU, representing an 11-fold difference between the two groups. Additionally we found a significant difference in the responses between the mock infected ferrets (group 5) and H1N1 infected ferrets (group 1 and 2) (P 0.0017). Comparison of lung MNCs in influenza and mock infected ferrets (group 5) showed no significant difference in the influenza-specific IFN-γ responses for mock (group 5) and H3N2 infected ferrets (group 3 and 4). Only two H3N2 infected ferrets were found to have influenza specific IFN-γ responses in lung MNCs, no responses were detected in remaining ferrets. Moderate positive correlations for all groups were found between the peak virus titre shed and the number of lung MNCs secreting influenza-specific IFN-γ (R +0.5775, P 0.0096), and the total amount of virus shed by ferrets and the number of lung MNCs secreting influenza-specific IFN-γ (R +0.5870, P 0.0082).

All virus infected ferrets (groups 1–4) showed a significant increase in the number of influenza-specific INF-γ secreting PBMCs when compared to the mock infected ferrets (group 5) at 14 dpi (H1N1 P 0.0002, H3N2 P 0.0004). No significant difference was observed between the number of PBMCs producing influenza-specific IFN-γ responses in the H1N1 and H3N2 infected groups (**Fig 4B**). These PBMC responses support the results seen in the longitudinal time course as measured by ELISA; there are similarities with both the influenza specific IFN-γ responses being produced over the course of the infection and number of cells responsible for producing influenza specific IFN-γ response as a result of infection with the two subtypes of influenza.

## Discussion

Influenza A virus infection can result in a spectrum of disease outcomes in humans, ranging from sub-clinical with seasonal strains to lethal with subtypes such as H5N1 and H7N9. The ability of seasonal influenza A viruses to continuously evolve antigenically means individuals can become infected on numerous occasions throughout their lives despite having immunity to previous strains. This antigenic drift also necessitates continuous updating of the trivalent and tetravalent vaccines. In this study our aims were to demonstrate the use of new reliable techniques to compare the kinetics and signatures of acquired immunity in ferrets challenged with low dose infections of seasonal influenza.

A low dose ferret model of infection for IAV can be considered more appropriate when attempting to model influenza infection as it represents a more ‘true to life’ dose of influenza[17]. We have previously demonstrated, using an H1N1 2009 pandemic virus, that there is an approximately 100-fold increase in the innate immune cell count in the nasal wash when infecting animals at a dose of 100 pfu, with a delay in peak cell count compared to infection with a high dose (10^6^ pfu). This suggests a delay in the activation of innate immune cell response found in the nasal wash which comprises of mostly neutrophils and monocytes/macrophages[32]. We have also shown that a lower challenge dose does not lead to reduced virus shedding, but instead leads to increased shedding both in terms of total virus shed over the course of the infection, and peak titre of shed virus[17]. Here we have illustrated that ferrets infected with a low dose (100 pfu) of H1N1 virus shed significantly more virus over a 14 days period compared to ferrets infected with an equivalent dose of H3N2 virus. This also correlates strongly with the peak shedding titre seen in the ferrets, with H1N1-infected ferrets having a higher peak shedding titre than animals infected with H3N2 virus. This is most likely due to a difference in replicative efficiency between the virus strains, as we did not observe a significant difference in the peripheral innate or adaptive immune responses to the viruses.

Comparison of the H1N1 and H3N2 influenza-specific IFN-γ time course in peripheral blood shows a similar pattern of responses in ferrets infected with either subtype. These results, combined with the PBMC ELISpot results, obtained at 14 dpi, suggest that the influenza-specific IFN-γ response as a marker of the cellular immune response seen in ferrets infected with H1N1 and H3N2 is not differentiated by sub-type and is instead comparable across both strains in the periphery. As established previously, the increase in the number of IFN-γ producing lymphocytes following challenge suggests an expansion of the numbers of influenza-specific T-cells as a consequence of progressive viral infection[33]. NK cells play an important role in controlling the virus in the early stages of infection[34]; therefore it is also possible that a fraction of these influenza-specific IFN-γ producing cells may be NK cells. Similar kinetics of influenza specific IFN-γ responses in PBMCs have been reported in pigs infected with a swine influenza (H1N2) virus[35]. This suggests that the influenza specific IFN-γ responses seen in the periphery do not vary significantly between seasonal strains or perhaps even host; however it is possible that the responses seen in a more clinically severe strain, such as H5N1 or H7N9, may be different. Although the responses observed in the periphery are not direct representations of the responses at the site of infection it is valuable to monitor the cellular immune response across a time course, especially in a model where only small volumes of blood can be collected. The key significant difference seen between the groups of infected ferrets was the number of influenza-specific IFN-γ producing cells seen in lung MNCs of ferrets infected with H1N1 compared to H3N2 infected ferrets and the mock-infected control group. It has previously been shown that A/California/04/2009 can be detected in the lung following low-dose intra-nasal infection[17]. This suggests that the influenza specific IFN-γ responses seen in the lungs of ferrets infected with H1N1 was due to virus causing infection in the lungs. In comparison the ferrets infected with H3N2 have either no or low influenza-specific IFN-γ responses. It has previously been reported that seasonal strains of H3N2 infrequently go to the lungs of ferrets[16, 31, 36–39], for example in one study H3N2 virus was delivered mostly by the intra-tracheal route using a high dose inoculum (10^6^ TCID_50_); despite this, very little evidence of H3N2 viral replication was seen in the lower respiratory tract[38]. These observations are in line with our findings of the minimal influenza specific IFN-γ responses seen in the lungs of ferrets infected with H3N2 virus.

This study demonstrates that it is possible to successfully evaluate cellular immune responses to seasonal influenza infection over a time-course in the ferret, and reveals that there are differences in the immune signatures induced by different influenza strains. By sequentially taking low volumes of heparinised blood samples from individual animals and detecting influenza-specific IFN-γ as measured by ELISA, we are able to form a picture of what is occurring in the periphery throughout infection. Furthermore, when the animals were sacrificed, we were able to quantify influenza-specific IFN-γ secreting cells in tissues of interest. While the use of the ELISA and ELISpot techniques is not unique, their use to examine influenza specific IFN-γ responses as a marker of the cellular immune response over a time course in the ferret model is. Using these complementary techniques, we have demonstrated that the responses to H1N1 and H3N2 infections in the lung were markedly different and correlated well with known tropisms of the virus strains used in this study. We believe this study enhances the applicability of the ferret model to study acquired immunity and vaccination strategies to influenza.

The current inactivated vaccines are poor inducers of T-cell responses. Cross-reactive T-cell responses are important in the protective response to novel vaccines, especially those based on internal virus antigens [40] and so the ability to measure these responses in the ferret model of influenza will become increasingly valuable. In the future we need to be able to measure both humoral and cellular responses in animal models as new vaccines are likely to require induction of both.
